# The Influence of Synthesis Method on the Local Structure and Electrochemical Properties of Li-Rich/Mn-Rich NMC Cathode Materials for Li-Ion Batteries

**DOI:** 10.3390/nano12132269

**Published:** 2022-06-30

**Authors:** Mylène Hendrickx, Andreas Paulus, Maria A. Kirsanova, Marlies K. Van Bael, Artem M. Abakumov, An Hardy, Joke Hadermann

**Affiliations:** 1 EMAT, Department of Physics, University of Antwerp, Groenenborgerlaan 171, 2020 Antwerp, Belgium; mylene.hendrickx93@gmail.com; 2 DESINe Group, Materials Chemistry, Institute for Materials Research (IMO-Imomec), Hasselt University, Agoralaan Building D and Imec, Division Imomec, and Energyville, 3590 Diepenbeek, Belgium; andreas.paulus@uhasselt.be (A.P.); marlies.vanbael@uhasselt.be (M.K.V.B.); an.hardy@uhasselt.be (A.H.); 3 Skolkovo Institute of Science and Technology, Center for Energy Science and Technology, Nobel Str. 3, 121205 Moscow, Russia; maria.kirsanova@skoltech.ru (M.A.K.); a.abakumov@skoltech.ru (A.M.A.)

**Keywords:** TEM, solution gel, coprecipitation, Li-ion battery, cathode, NMC

## Abstract

Electrochemical energy storage plays a vital role in combating global climate change. Nowadays lithium-ion battery technology remains the most prominent technology for rechargeable batteries. A key performance-limiting factor of lithium-ion batteries is the active material of the positive electrode (cathode). Lithium- and manganese-rich nickel manganese cobalt oxide (LMR-NMC) cathode materials for Li-ion batteries are extensively investigated due to their high specific discharge capacities (>280 mAh/g). However, these materials are prone to severe capacity and voltage fade, which deteriorates the electrochemical performance. Capacity and voltage fade are strongly correlated with the particle morphology and nano- and microstructure of LMR-NMCs. By selecting an adequate synthesis strategy, the particle morphology and structure can be controlled, as such steering the electrochemical properties. In this manuscript we comparatively assessed the morphology and nanostructure of LMR-NMC (Li_1.2_Ni_0.13_Mn_0.54_Co_0.13_O_2_) prepared via an environmentally friendly aqueous solution-gel and co-precipitation route, respectively. The solution-gel (SG) synthesized material shows a Ni-enriched spinel-type surface layer at the {200} facets, which, based on our post-mortem high-angle annual dark-field scanning transmission electron microscopy and selected-area electron diffraction analysis, could partly explain the retarded voltage fade compared to the co-precipitation (CP) synthesized material. In addition, deviations in voltage fade and capacity fade (the latter being larger for the SG material) could also be correlated with the different particle morphology obtained for both materials.

## 1. Introduction

Nowadays lithium-ion battery technology is still the best choice for electrochemical energy storage, facilitating the transition from polluting fossil fuels to renewable sources. Lithium- and manganese-rich nickel manganese cobalt oxides (LMR-NMCs) have been extensively studied as promising positive electrode (cathode) materials for rechargeable lithium-ion batteries due to their high initial specific capacities exceeding 280 mAh/g [1,2,3], high thermal stability [2,4] and low costs [5]. However, despite these high capacities, their commercialisation is still hampered by significant voltage fade and voltage hysteresis deteriorating the specific energy [1,6]. These issues originate from the crystal and electronic structure of LMR-NMCs. Cationic redox reactions (i.e., Co^3+^ → Co^4+^ and Ni^2+^ → Ni^3+^ → Ni^4+^) and reversible oxygen redox processes contribute to the capacity of LMR-NMCs [6]. However, when lithium ions leave the host structure upon charging, transition metal (TM) cation migration occurs, which is partially irreversible. Upon cycling, these defects accumulate, causing a gradual transition of the layered *C2*/*m* structure to a spinel-type structure and eventually to a rock-salt-type structure, where the transition metals and lithium are randomly distributed over the cation positions. These structural changes are closely related to the oxygen redox processes, with the partial irreversibility of the cation migration causing voltage fade [7]. Voltage hysteresis, for its part, appears to originate from kinetic limitations in the electron transfer, as recently published by Li et al. [8].

Besides stoichiometry, the electrochemical performance (including voltage and capacity fade) also strongly depends on the selected LMR-NMC synthesis strategy, which steers the material properties, such as the particle morphology (size and shape), phase purity, crystallinity, and cation disorder [9]. Many synthetic methods have been developed to control and improve the electrochemical performance of the cathode materials incorporated in Li-ion batteries. At the beginning of the discovery of LMR-NMCs, these materials were synthesized through a high-temperature heat treatment that generally leads to excessive particle growth and poor performance. These days, among the various methods, solution-gel (SG) and co-precipitation (CP) routes are commonly applied [9,10,11] to synthesize multimetal oxide cathode materials. Solution-gel methods are very popular due to their ability to achieve good stoichiometry and to mix reactants homogeneously at an atomic level. On the other hand, co-precipitation methods are very suitable to tune the particle morphology and can be more easily upscaled.

Therefore, in this paper, we comparatively assess the structure and morphology of Li_1.2_Ni_0.13_Mn_0.54_Co_0.13_O_2_ compounds prepared using a SG and CP synthesis route and study the correlation of the structural differences with the electrochemical properties.

## 2. Materials and Methods

### 2.1. Synthesis

#### 2.1.1. Aqueous Solution-Gel Method

The first step of the aqueous solution-gel synthesis typically involves dissolving metal ions in water by complexation using a chelating agent. Citric acid is often used as a chelating agent after deprotonation by increasing the pH: due to its three carboxylic groups per molecule, it can form bridges between different metal ions. The next step is the solvent evaporation that results in the formation of a viscous gel by the linking of different metal ions and ligands via hydrogen bridges. Finally, the thermal decomposition of the multimetal ligand gel during the calcination and crystallization step at an elevated temperature leads to highly crystalline particles [9,10].

For this work, LMR-NMC with the composition Li_1.2_Ni_0.13_Mn_0.54_Co_0.13_O_2_ was prepared via an aqueous solution-gel method by using aqueous LiNO_3_ (LiNO_3_ precursor; Sigma Aldrich, 99%), Ni(NO_3_)_2_ (Ni(NO_3_)_2.6_H_2_O precursor; Sigma Aldrich, ≥97%), Co(NO_3_)_2_ (Co(NO_3_)_2.6_H_2_O precursor; Alfa Aesar, ≥98%), and Mn(NO_3_)_2_ (Mn(NO_3_)_2_.xH_2_O precursor; Sigma Aldrich, 98%) monometal precursors. Consecutively, citric acid (C_6_H_8_O_7_, Sigma Aldrich, 99%) was added to an aqueous mixture containing stoichiometric amounts of the aforementioned metals in a 1.5:1 molar ratio of citric acid:TM (TM = transition metal ion). A lithium excess of 5 molar% was used to compensate for the loss of volatile Li-containing products at elevated calcination temperatures. After stirring for about 15 min at room temperature to dissolve the citric acid, the pH was increased to ~7 by dropwise addition of NH_3_ (Merck, extra pure, 32%). The resulting mixture was refluxed at 80 °C for 1 h. Afterwards, the mixture was placed in an airflow oven for gelation under atmospheric conditions at 60 °C overnight. The obtained viscous gel was pre-calcined under atmospheric conditions at 200 °C in an airflow oven. The ground pre-calcined, brown-colored powder was calcined and crystallized in a tube furnace at a heating rate of 10 °C/min up to 900 °C with an isothermal period of 12 h. The heating up to 900 °C was performed under a dynamic dry air atmosphere (AirLiquide, α1, 99.999%). The isothermal period at 900 °C was performed under a zero-flow rate of dry air. It should be noted that the presence of nitrates in the precursor implies that a combustion reaction was involved during the calcination step, despite the fuel-oxidizer ratio not being intentionally optimized. After naturally cooling down to room temperature, the resulting product was ground and stored in closed glass vials in an Argon glovebox for further use.

#### 2.1.2. Aqueous Co-Precipitation Method

The aqueous co-precipitation synthesis involves two main steps: in the first step, an insoluble product is formed containing the different metal ions. The precipitation of the insoluble particulate matter is initiated by the simultaneous addition of dissolved metal salts and co-precipitating agent at controlled pH. The precipitate is typically washed and centrifuged multiple times to remove the precipitating agent’s metal cation, the metal salt’s counterion (particularly when starting from sulphate precursors), and non-reacted starting products. The materials are dried after washing to remove the remaining water. In the second step, the precursors are mixed with a lithium source and calcined at elevated temperatures to produce the crystalline cathode material [11].

For this work, LMR-NMC with the composition Li_1.2_Ni_0.13_Mn_0.54_Co_0.13_O_2_ was prepared by a carbonate co-precipitation method, where the precursor was a mixed Ni, Mn, Co carbonate phase [12]. The co-precipitation reaction was performed in a stirred Globe batch reactor (Syrris Ltd., Royston, UK) under N_2_ atmosphere at 60 °C. Stoichiometric amounts of NiSO_4_.7H_2_O, CoSO_4_.7H_2_O, MnSO_4_.H_2_O (all—Ruskhim, ≥99%.) were dissolved in distilled water. Beforehand, the water of hydration/crystallization content was determined using TGA measurements (Netzsch STA 449 F3 Jupiter, Germany). The aqueous solution, with a total Ni+Mn+Co metal ion concentration of 2 mol/L, was added dropwise simultaneously with a 2 mol/L Na_2_CO_3_ (Ruskhim, 99.5%) solution, at a fixed rate of 1 mL/min, into the reactor preheated at 60 °C. The reaction was performed under continuous stirring, whilst adjusting the pH to stay constant at 7.5 by addition of dilute NH_3_ (Khimmed, puriss. p.a. ≥ 25% NH_3_ in H_2_O) or H_2_SO_4_ (Khimmed, 98 w.% H_2_SO_4_ in H_2_O). After six hours, the stirrer and temperature controller were automatically switched off. The resulting precipitate was washed with distilled water and centrifuged three times for three minutes at 5700 rpm. After drying at 75 °C overnight in a vacuum furnace, the mixed carbonate NMC precursor was obtained, which was dry mixed with Li_2_CO_3_ (Sigma Aldrich, anhydrous ≥ 99%). An excess of 10 mol% Li_2_CO_3_ was added over the stoichiometric amount to compensate for lithium loss during calcination. After manual grinding in a mortar, the samples were consecutively calcined at a heating rate of 5 °C/min up to 500 °C with an isothermal period of 5 h in a tube furnace. Then they were naturally cooled down, ground again for 15 min in an agate mortar and pestle and calcined at a heating rate of 5 °C/min up to 900 °C with an isothermal period of 12 h in a tube furnace. The heating up to 500 °C was performed under a dynamic dry air (AirLiquide, α1, 99.999%) atmosphere. The isothermal period at 500 °C and the full calcination at 900 °C were performed under static ambient air. The resulting product LMR-NMC was ground after naturally cooling down to room temperature and stored in closed glass vials sealed with parafilm under ambient air for further use.

### 2.2. Structural Characterization

The samples were characterized by X-ray powder diffraction (XRPD), scanning electron microscopy (SEM), selected area electron diffraction (SAED) and high-angle annular dark-field scanning transmission electron microscopy (HAADF-STEM), coupled with energy-dispersive X-ray analysis (EDX).

The XRPD patterns were recorded on a Huber G670 Guinier diffractometer making use of Cu Kα1 radiation. The SEM images were collected using a Thermo Fisher Quanta FEG 250 microscope at 20 kV. The SAED patterns, HAADF-STEM images and STEM-EDX maps were acquired using a Thermo Fisher Titan 80–300 “cubed” microscope equipped with a Super-X detector and operated at 300 kV. The pristine samples for SAED, STEM and STEM-EDX were made by crushing and dispersing the powder in ethanol using an ultrasonic bath. The powder used for the particle size investigation was not crushed, only dispersed in ethanol using an ultrasonic bath. A few droplets of the obtained suspension were deposited on a Cu grid covered with a holey carbon layer. The charged and cycled samples were prepared under argon atmosphere in a glovebox. After charging or cycling, coin cells were disassembled in an argon-filled glovebox, followed by washing the cathode punches three consecutive times with dimethyl carbonate (DMC). Next, the powder was scratched from the cathode punch and crushed, after which a copper grid with a holey carbon layer was dipped into the dry powder. Finally, the samples were transferred to the TEM using a GATAN vacuum transfer holder to exclude contact with ambient atmosphere.

### 2.3. Electrochemical Characterization

The SG-NMC and CP-NMC active materials were electrochemically characterized in a coin cell configuration. The procedure to prepare a positive electrode coating started with ball milling a mixture of carbon black (Imerys, Super C65), polyvinylidene difluoride (PVDF, Alfa Aesar) in N-methyl pyrrolidone (NMP, Alfa Aesar) and the active material (10:10:80 weight ratio) to obtain a slurry. Next, the slurry was tape casted on an aluminium current collect, aiming at a wet-coating thickness of about 150 µm. This was followed by a drying step in a vacuum oven at 110 °C. A coin cell contained a stacking of the positive electrode, a polypropylene/polyethylene/polypropylene trilayered membrane (Celgard 2400) as the separator and Li metal (Sigma Aldrich, 99.9% trace metals purity) as the negative electrode. 1M LiPF6 in EC:DMC (Sigma Aldrich, 50/50 (*v*/*v*)% ethylene carbonate:dimethyl carbonate) served as the electrolyte. The galvanostatic cycling protocol was composed of two formation cycles at a C-rate of C/20, followed by fifty cycles at C/10, both performed within the 3.0 V–4.6 V vs. Li^+^/Li interval. The C-rate was calculated based on an assumed theoretical capacity of 250 mAh/g. Galvanostatic cycling was preceded by a relaxation time of 24 h after coin cell assembly. The galvanostatic cycling measurements were performed at least in threefold to confirm the reproducibility.

## 3. Results

### 3.1. Structural Characterization

**Figure 1:**
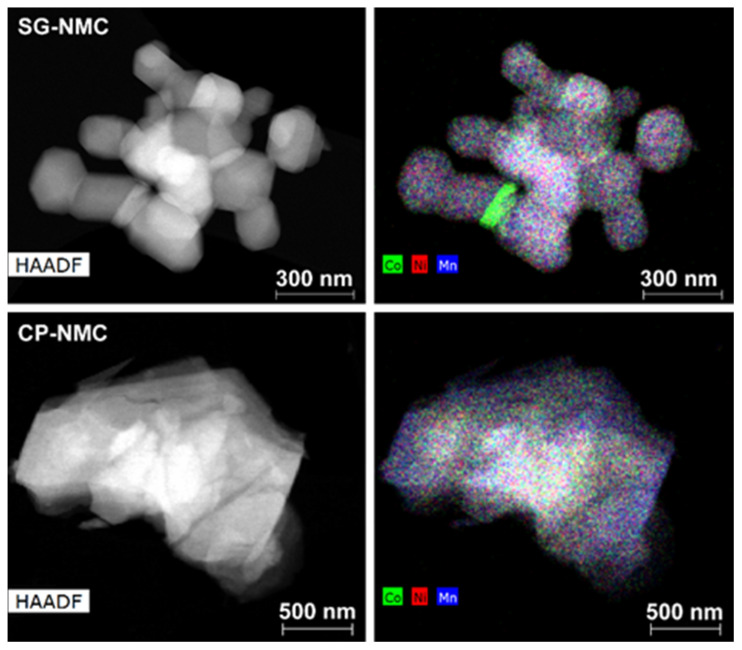
The HAADF-STEM image (**left**) together with the mixed Co/Ni/Mn element map (**right**) of representative particles from the Li_1.2_Ni_0.13_Mn_0.54_Co_0.13_O_2_ samples prepared with the solution-gel (**top**) and co-precipitation (**bottom**) method. The mixed element map of the SG-NMC sample reveals the presence of particles with an additional Co-rich phase.

Representative low magnification HAADF-STEM images and EDX maps of the Li_1.2_Ni_0.13_Mn_0.54_Co_0.13_O_2_ samples synthesized by the solution-gel (SG-NMC) and co-precipitation (CP-NMC) methods are shown in Figure 1. Additional SEM images of both samples are given in the supporting information (Figure S1). The particles of the SG-NMC sample range between 100 nm and 480 nm with an isotropic shape and pronounced facets, while the particles from the CP-NMC sample have a size from 250 nm to 2 μm and have an irregular shape and a rough surface. The EDX maps show that for the bulk of the main phase, the transition metals are homogeneously distributed. The average composition of both samples was determined from 45–60 point spectra distributed over 20–30 crystallites and is in good agreement with the expected one, with transition metal cation contents of Ni_0.15(1)_Mn_0.52(2)_Co_0.13(1)_ for the SG-NMC sample and Ni_0.14(2)_Mn_0.53(3)_Co_0.13(2)_ for the CP-NMC sample. In the case of the SG-NMC sample, a Co-rich admixture phase is also present; however, the amount of the Co-rich phase is negligible, which is confirmed by the XRPD patterns shown in the supporting information (Figure S2). The formation of a Co-rich phase for SG-NMC can be due to a non-optimal calcination of the precursor gel.

**Figure 2:**
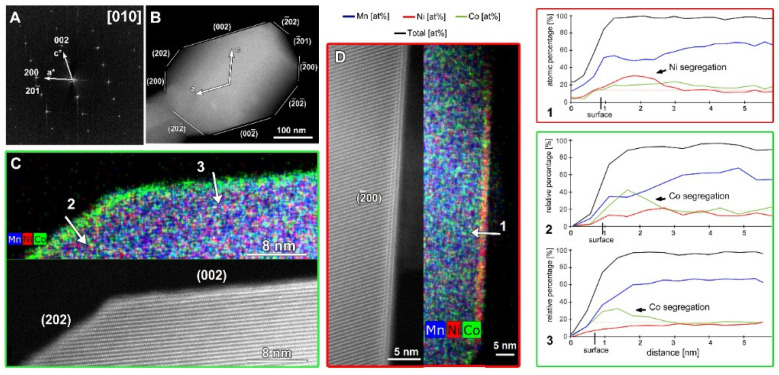
The SAED pattern (**A**) and low magnification HAADF-STEM image (**B**) of the particle of SG-NMC taken along the [010] zone-axis. (**C**) Atomic resolution HAADF-STEM image with the corresponding mixed (Mn, Ni, Co) EDX map showing the (202) and (002) facets. (**D**) Atomic resolution HAADF-STEM image with the corresponding mixed (Mn, Ni, Co) EDX map showing the ($\bar{2}$00) facet. The arrows marked with numbers 1, 2 and 3 correspond to the line profiles that are shown on the right.

Representative SAED patterns and HAADF-STEM images of the SG-NMC and CP-NMC samples are included in the supporting information (Figures S3 and S4) and show that, for both samples, the crystal structure of the bulk material corresponds to the layered *C2*/*m* structure with a honeycomb ordering between the TM and Li atoms within the mixed TM-Li layers. However, the atomic resolution HAADF-STEM images taken from the SG-NMC sample (Figure 2) also reveal a structural modification at specific surface planes, which is not present in the CP-NMC sample. The (200) facets have a Ni-rich surface layer with a homogeneous thickness of ~1.5 nm, while other surface facets do not have such Ni-rich composition. On the contrary, the (002) and (202) planes exhibit Co-segregation. The Ni/Co-segregation is supported by the line profiles taken perpendicular to these different facets. The line profiles show the relative percentages of only Ni, Mn and Co, which for Ni_0.13_Mn_0.54_Co_0.13_ agree with the nominal ratio Ni:Mn:Co = 16.25:67.5:16.25. The maximum observed sum of Ni, Co and Mn (black curve) is set to 100%, and all other values are relative to this maximum. At the surfaces, the total amount of transition metals decreases gradually rather than abruptly. Lu et al. [13] demonstrated that lithium-containing oxides suffer from radiation damage by high-energy electron beams that involves the creation of lithium vacancies and the migration of transition metals. HAADF-STEM images taken before and after the acquisition of the EDX map (see Figure S5) clearly show that the increased intensity at the (200) facets, corresponding to the Ni-enriched surface layer, is also present before the EDX acquisition. This proves that the Ni- and Co-segregation is not induced by the long exposure of the electron beam during the acquisition of the EDX map.

**Figure 3:**
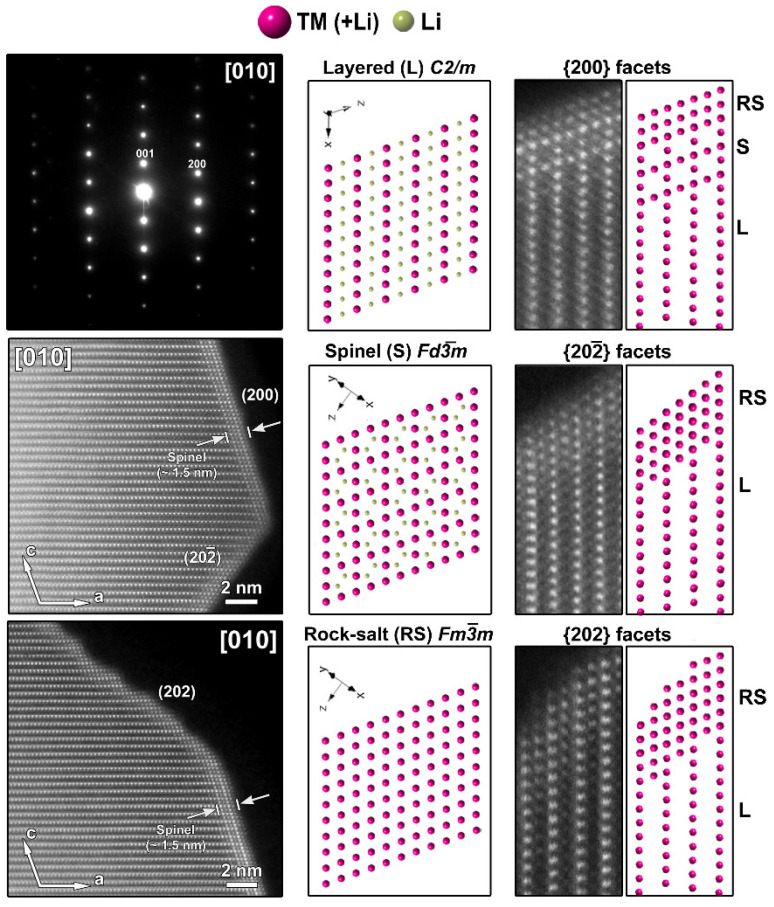
**Left**: SAED pattern and two HAADF-STEM images taken along the [010] zone-axis from the pristine SG-NMC sample. The HAADF-STEM images clearly show the spinel structure at the (200) surface and a rock-salt-type structure at the (202) and ($20\bar{2}$) surfaces. **Middle**: the models of the layered, spinel and rock-salt structure projected along the [010] orientation, which is equivalent to the [$\bar{1}\bar{1}0$ ] orientation in the spinel and rock-salt structures. To simplify the models, only TM and Li are shown. **Right**: Close-up HAADF-STEM images of the (200), (202) and ($20\bar{2}$ ) surfaces are shown together with the simulated structures. In the HAADF-STEM image, only the TM positions will be clearly visible and therefore also only the TM cations are shown on the schemes. The surface of the (200) facet consists of a spinel layer with two rock-salt-type layers on top. The surface of the ($20\bar{2}$ ) and (202) facets correspond to a rock-salt-type structure.

The atomic resolution HAADF-STEM image in Figure 3 shows the two types of surface modification together with the SAED pattern along the [010] zone-axis of the SG-NMC sample. The intensity of the atom columns in the HAADF-STEM images is proportional to the squared atomic number of the elements (I~Z^2^). Therefore, the bright dots in the HAADF-STEM images correspond to the transition metal atom columns, while the signals from atom columns of lithium and oxygen are too weak to be clearly visualized. Based on the HAADF-STEM image, the surface of the (200) facets is formed by a spinel-type layer with one atomic layer with a disordered distribution of the transition metals and lithium atoms on top. The higher intensity at the surface of the (200) facets can be associated with the higher amount of transition metals present in a spinel structure: the spinel structure Li(TM)_2_O_4_ contains a higher concentration of TM relative to oxygen and lithium than the targeted Li_1.2_(TM)_0.8_O_2_. In contrast to the ordered spinel structure at the (200) facets, the surface layers at the (202) and ($20\bar{2}$) facets tend to form a rock-salt-type structure with a disordered placement of transition metals and lithium atoms over the cation positions. The higher intensity observed at the (202) and ($20\bar{2}$) surfaces agrees with the higher density of the transition metals in the rock-salt structure. Combining our results, we can conclude that only the (200) facets form a Ni-rich spinel-type surface layer, while Co-segregation is present on the (002) facets and the rock-salt-type surface of the (202) facets. The ($20\bar{2}$) facets have a similar rock-salt type surface to the (202) facets but have no Co-segregation (Figure S6).

**Figure 4:**
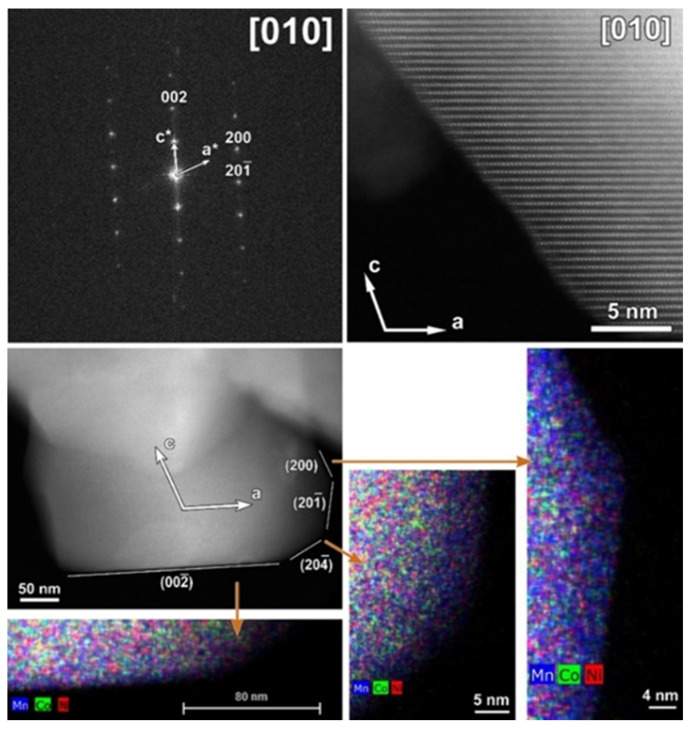
**Top**: [010] SAED pattern of the CP-NMC indexed in the *C2*/*m* space group and corresponding high-resolution HAADF-STEM image (white arrows in the SAED pattern and HAADF-STEM image indicate the direction of reciprocal and direct crystallographic axes, respectively). **Bottom**: Low-magnification HAADF-STEM image with denoted facets and axes and mixed (Mn, Co, Ni) EDX maps of a representative particle from the pristine CP-NMC sample. The mixed EDX maps show the absence of Ni- and Co- segregation at the (200) and (00$\bar{2})$ facets, respectively.

The typical atomic resolution [010] HAADF-STEM image of the CP-NMC illustrates the perfect arrangement of TM columns without positional disordering or any other structural modifications. Although particles of CP-NMC material in general do not have a polygonal shape, one of them with labeled crystal facets is shown in Figure 4. As illustrated by corresponding EDX element maps, no segregation of Co or Ni was observed at any of the facets.

### 3.2. Electrochemical Characterization

**Figure 5:**
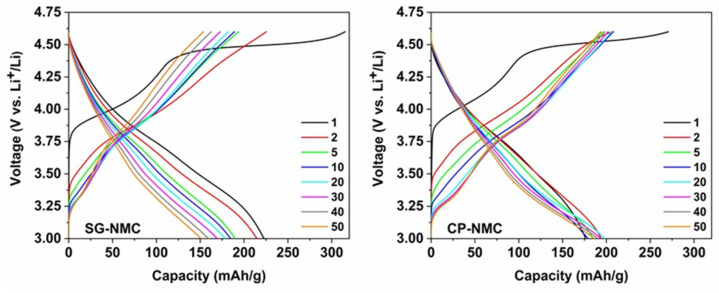
The charge-discharge curves of the Li_1.2_Ni_0.13_Mn_0.54_Co_0.13_O_2_ sample synthesized by the solution-gel (**left**) and the co-precipitation (**right**) method. The two formation cycles were performed at a C-rate of C/20, the subsequent cycles at C/10.

The electrochemical properties of the SG-NMC and CP-NMC samples were comparatively assessed via galvanostatic charge/discharge cycling. The materials were exposed to 50 charge/discharge cycles at a C-rate of C/10 in the voltage interval of 3.0 V–4.6 V vs. Li^+^/Li preceded by two formation cycles at C/20. The corresponding charge/discharge curves are plotted in Figure 5.

**Figure 6:**
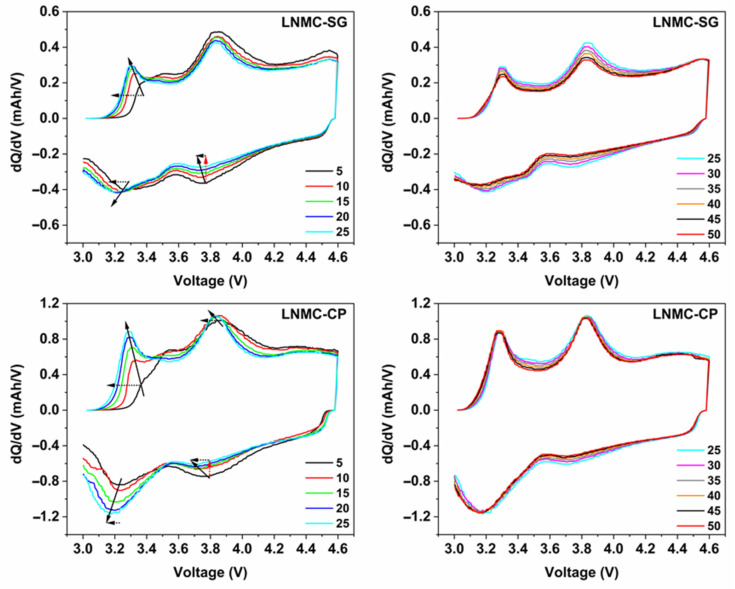
Differential capacity (dQ/dV) vs. voltage profiles derived from selected charge/discharge curves for SG-NMC (**top**) and CP-NMC (**bottom**). Smoothing has been performed using the Percentile Filter (Percentile: 50; Points of Window: 10) using the origin software. Voltage fade and capacity fade are indicated by black dotted and red dotted arrows, respectively. The solid line arrow indicates, by approximation, the shift in peak maxima as a function of the cycle number.

**Figure 7:**
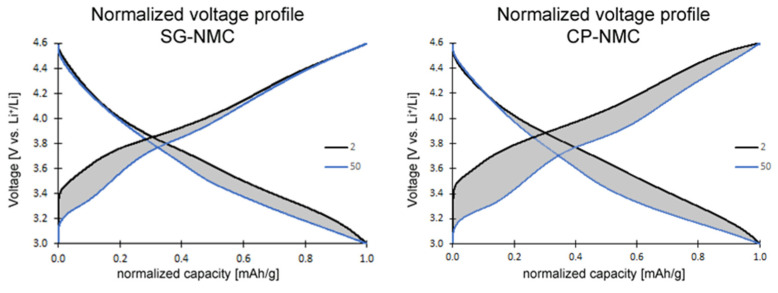
Normalized voltage profiles of the SG-NMC (**left**) and CP-NMC (**right**) samples. The charge and discharge capacity of the 2nd and 50th cycles are normalized to illustrate the voltage fade. The average voltage decreased by approximately 88 mV and 173 mV for the SG-NMC and CP-NMC samples, respectively.

The slope region of the initial charging step, centered around 3.8 V vs. Li^+^/Li (as derived from the dQ/dV vs. V profiles in Figure 6), involves cation oxidation (i.e., Ni^2+^ → Ni^3+/4+^ and Co^3+^ → Co^4+^). This is followed by a voltage plateau at about 4.5 V vs. Li^+^/Li attributed to oxygen redox processes eventually resulting in oxygen release. The observed voltage fade is higher for the CP-NMC sample than for the SG-NMC sample, which is indicated by the normalized voltage profiles shown in Figure 7. Voltage decay is observed in both the charge and discharge process, and the average voltage decay of SG-NMC and CP-NMC corresponds to approximately 88 mV and 173 mV, respectively, for 50 charge/discharge cycles.

**Figure 8:**
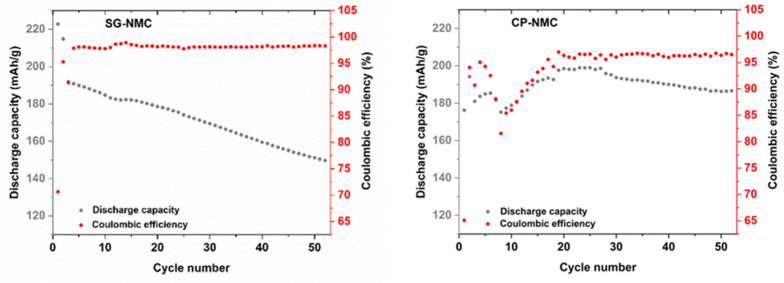
Discharge capacity and Coulombic efficiency vs. cycle number for SG-NMC (**left**) and CP-NMC (**right**).

The capacity fade of the SG-NMC sample of about 70 mAh/g between the initial and the 50th cycle is much more pronounced than that of the CP-NMC sample. The discharge capacities and the corresponding capacity fade can be better visualized in Figure 8, which depicts the discharge capacity and the Coulombic efficiency versus the cycle number. Upon increasing cycle number, the discharge capacity of the SG-NMC sample shows a constant decay, while the Coulombic efficiency fluctuates around 98%. This implies that the capacity fade for SG-NMC cannot be fully ascribed to an irreversible process occurring exclusively during charging or discharging within the same cycling step, but mainly to a continuous degradation of the material during cycling. For the CP-NMC sample, a different electrochemical behavior is observed. The significantly lower initial discharge capacity for CP-NMC compared to SG-NMC could be attributed to the higher Li-excess used during the synthesis of the former [14]. The discharge capacities of the subsequent 5–10 charge/discharge cycles for CP-NMC show a major decrease followed by a steady increase in higher values than for SG-NMC, until cycle number 25. For the synthesis of CP-NMC a higher Li-excess was used than for SG-NMC. For over-lithiated Li-rich NMC, the transition metals are found by Luchkin et al. [15] to be in a higher oxidation state. This hampers the capacity of the initial cycles, as these transition metals cannot all be further oxidized. The capacity recovery after 10 cycles for CP-NMC originates from the progressing activation of anionic redox (oxidation of O^2−^ to O^n−^ (*n* < 2)), resulting in a reduction in transition metals. These reduced transition metals (we refer to Mn^3+^/Mn^4+^ redox couple, as evidenced by the dQ/dV vs. V profiles in Figure 6) can contribute to the capacity in subsequent cycles. This process occurs gradually depending on the propagation of the activation front into the agglomerates of the co-precipitated material. How this effect evolves during cycling is difficult to predict as it depends on the microstructure and porosity of agglomerates [15]. For higher cycle numbers, a decrease in the discharge capacity is observed. Until cycle number 25, the Coulombic efficiency for CP-NMC shows the same trend as the discharge capacity; this means that as the discharge capacity decreases, the same is valid for the Coulombic efficiency. This implies that the decrease in discharge capacity between cycle numbers 5 and 10 can be most certainly ascribed to an irreversible process during either the charging or discharging step. For cycle numbers above 25, the Coulombic efficiency remains more or less constant at around 97%.

In order to gain insight into the different electrochemical behavior of the SG-NMC and CP-NMC samples, the differential capacity (dQ/dV) vs. voltage profiles derived from the galvanostatic charge/discharge data are shown in Figure 6.

For both samples, the oxidation peak at about 3.2 V is related to the oxidation of Mn^3+^ to Mn^4+^, while the oxidation peak at around 3.8 V is attributed to the oxidation of Ni^2+^ to Ni^3+^ and Ni^4+^ and the oxidation of Co^3+^ to Co^4+^ [7,16,17,18]. Similarly, for the discharge process, the reduction peak between 3.6 V and 3.8 V vs. Li^+^/Li results from the Ni^4+^/Ni^2+^ redox couple and, to a lesser extent, from the Co^4+^/Co^3+^ redox couple. The reduction peak at around 3.2 V corresponds to the Mn^4+^/Mn^3+^ redox couple. The manganese redox chemistry is strongly correlated with the anionic redox chemistry of the oxygen sublattice [7,17,19].

The lowering of the (integrated) intensity of the reduction peak associated with the Ni^4+^/Ni^2+^ redox couple upon increasing cycle number and the simultaneous increase of the (integrated) peak intensity of the reduction peak attributed to the Mn^4+^/Mn^2+^ redox couple strongly indicates the transformation from the initial layered structure to a (disordered) spinel-type structure [7,16,17,20]. The increase of the Mn^4+^/Mn^3+^ reduction peak goes along with the increase in (integrated) peak intensity of the oxidation peak associated with the Mn^3+^/Mn^4+^ redox couple.

The lower accessibility of the Ni^4+^/Ni^2+^ redox couple for the spinel-type structure can be ascribed to the partial nickel migration to tetrahedral interstices and the lithium layer. During the first 25 charge/discharge cycles, the structural transformation to a spinel-type structure seems to occur more prominently for CP-NMC compared to SG-NMC, as suggested by the faster decay of the peak related to Ni^4+^/Ni^2+^ and a faster increase in the peak related to Mn^4+^/Mn^2+^. The faster irreversible structural transformations agree with the drop in Coulombic efficiency and discharge capacity for the CP-NMC sample in the cycle number window discussed above. For cycle numbers above 25, the (integrated) peak intensities for both reduction peaks remain constant, indicating a less prominent contribution of occurring structural transformations to the capacity for the initial 25 charge/discharge cycles. Anionic redox chemistry also partially accounts for the capacity delivered resulting in the oxidation peak at around 3.8 V and the reduction peak at about 3.2 V [19]. This explains why, specifically for the CP-NMC sample, the (integrated) peak intensity for the oxidation peak at around 3.8 V remains high, while the reduction peak for the Ni^4+^/Ni^3+^ and Ni^3+^/Ni^2+^ redox couples at 3.7 V is to a large extent flattened.

Voltage fade was comparatively assessed for both the SG-NMC and CP-NMC samples and is indicated by arrows on the differential capacity curves for the initial 25 cycles. From the results presented in this section, we suggest that the surface spinel-type layer at the above-mentioned facets, present for the SG-NMC sample, might retard the progressing voltage fade, but not completely prevent its occurrence. This hypothesis is also supported by our TEM study on the cycled SG-NMC sample as explained in the next section.

### 3.3. Structural Characterization after Galvanostatic Cycling

To gain understanding of the effect of extended galvanostatic cycling on surface modification layers and how differences in the observed electrochemical properties can be related to the structural differences, the same TEM study has been performed on the cycled SG-NMC and CP-NMC sample after 50 cycles.

**Figure 9:**
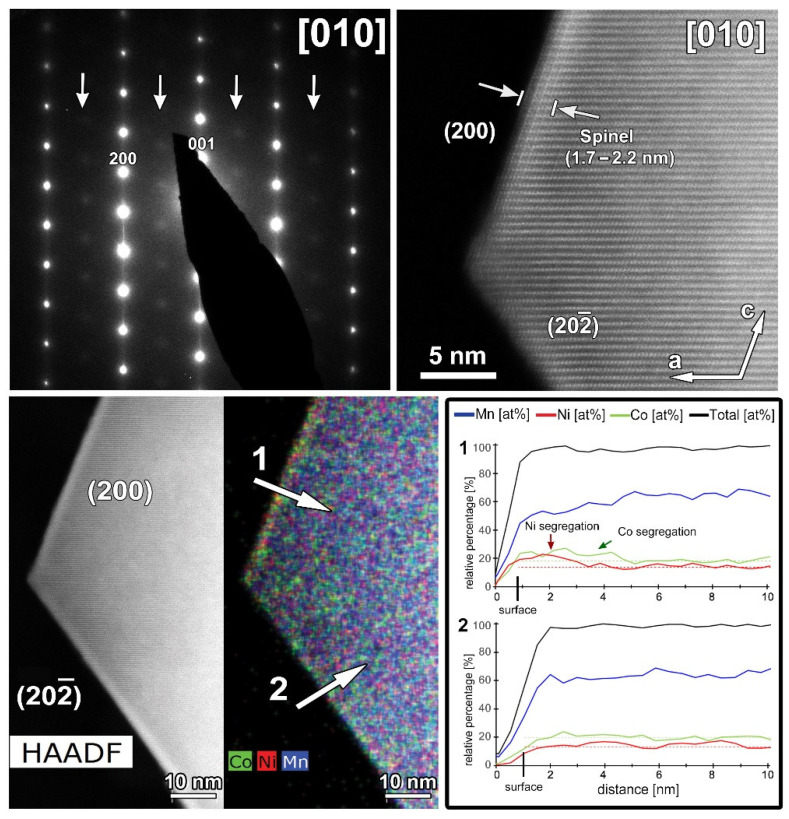
The cycled SG-NMC sample. **Top left**: SAED pattern along the [010] orientation. The arrows indicate extra diffuse intensities that can only be indexed by a spinel-type structure. **Top right**: high-resolution HAADF-STEM image along the [010] orientation showing a thickness increase of the surface spinel-layer at the (002) facet. **Bottom left**: HAADF-STEM image and corresponding EDX map, given in counts. The EDX map shows Ni- and Co-segregation at the (200) facet, and slight segregation of Co at the ($20\bar{2}$) facet. **Bottom right**: line profiles are taken perpendicular to the (200) (i.e., line profile 1) and ($20\bar{2}$ ) (i.e., line profile 2) facets. The line profile of the (200) facet confirms the segregation observed in the EDX map and exposes the small differences between the concentration at the surface and bulk. However, the line profile of the ($20\bar{2}$ ) facet does not show clear segregation.

**Figure 10:**
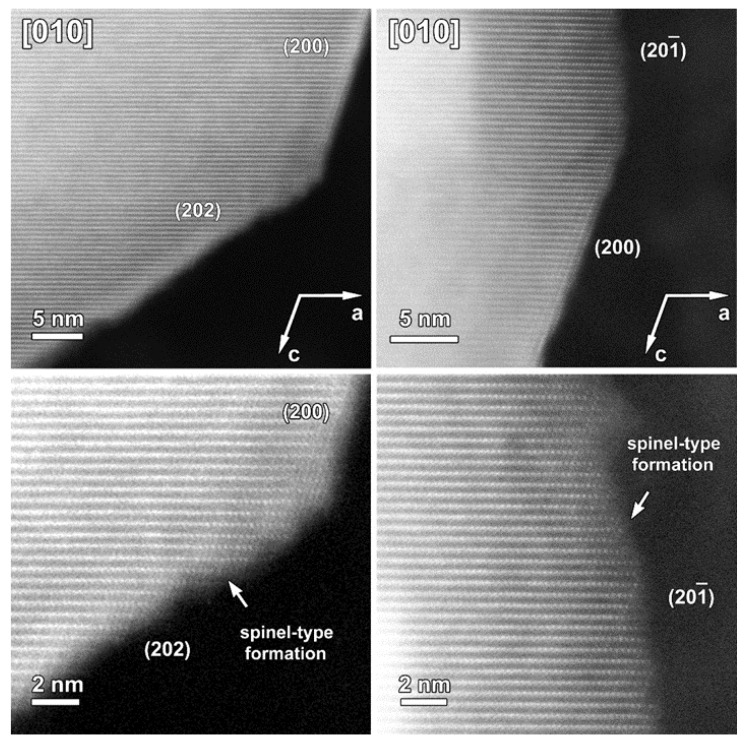
HAADF-STEM images of one particle of the cycled SG-NMC sample taken along the [010] orientation. Bottom close-up images show the structural degradation towards a spinel-type structure. The structural degradation mainly occurred at the facets (i.e., {202} and {$20\bar{1}$}) that do not contain such initial spinel-type surface modification as the {200} facets.

A representative HAADF-STEM image, SAED pattern and EDX maps recorded along the [010] zone-axis for the cycled SG-NMC sample are shown in Figure 9. The SAED pattern shows extra diffuse reflections that can only be indexed by a spinel structure, indicating the presence of a spinel-type phase. The SAED pattern is taken from the entire particle, including the spinel structure at the surface. The contribution of the spinel structure at the {200} facets is very small compared to the entire volume of the particle and is therefore negligible in the SAED pattern (otherwise reflections would also appear in the SAED patterns of the pristine sample, representative pattern in Figure 3). Since these reflections only appear in the cycled sample, they can be associated with structural degradation to a spinel-type phase. The HAADF-STEM image, taken from the same particle, shows that the spinel structure at the surface is preserved upon cycling but that the thickness is increased to approximately 1.7–2.2 nm. The diffuse reflections indicated by the arrows in the SAED pattern indicate a significant amount of conversion from the initial layered structure to a spinel-type structure. The minor increase in the thickness of the spinel-type surface layer at the {200} facets is unlikely to account for the appearance of the extra reflections. Indeed, the atomic resolution HAADF-STEM image along the [010] orientation (Figure 10) illustrates that the structure degradation mainly occurs at the other facets, specifically the (202) and ($20\bar{1}$) facets, which supports our hypothesis that the spinel-type surface layer at the {200} facets slows down the structure degradation at these specific facets. Furthermore, to investigate the element distribution, line profiles were taken from the (200) and ($20\bar{2}$) facets (Figure 9) that show a change in segregation of metal cations. In the EDX map, it seems that at both facets segregation is present; however, only the line profile perpendicular to the (200) facet clearly shows segregation. While in the pristine sample, only Ni-segregation was observed at the (200) facets (line profile 1 in Figure 2), both Ni- and Co-segregation are observed in the discharged sample (line profile 1 in Figure 9). This means that upon cycling, Co has segregated towards the (200) surface. To compose the Co-segregation towards the surface, the Ni excess at the surface might have partially dissolved upon cycling. This can occur since the highly reactive Ni^4+^ ions at the particle surface can react with the electrolyte at high voltages (>4.4 V vs. Li+/Li) [21].

**Figure 11:**
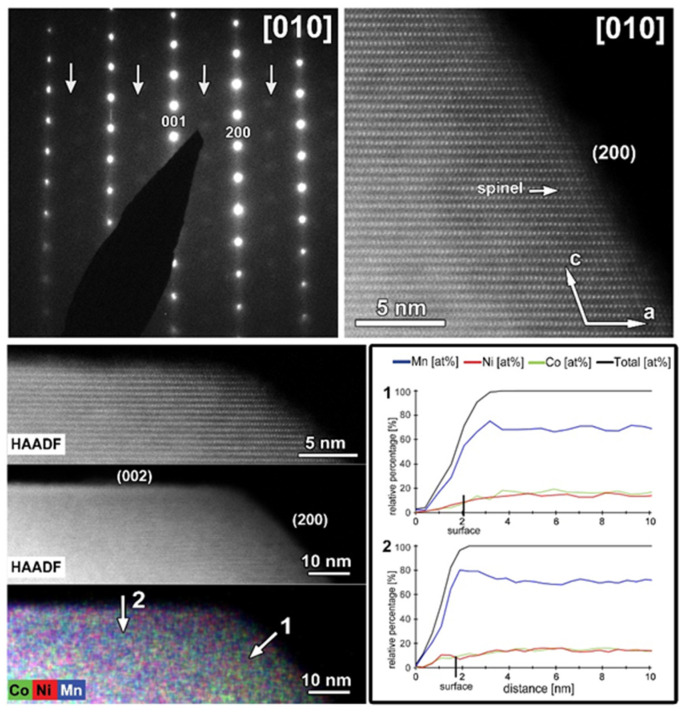
The cycled CP-NMC sample. **Top left**: SAED pattern along the [010] orientation. The arrows indicate extra diffuse intensities that can only be indexed by a spinel-type structure. **Top right**: high-resolution HAADF-STEM image along the [010] orientation showing the formation of a spinel-type phase at the surface. **Bottom left**: HAADF-STEM image and corresponding EDX map, given in counts. The EDX map shows no Ni- and Co-segregation, as expected for this sample. **Bottom right**: line profiles are taken perpendicular to the (200) (i.e., line profile 1) and (002) (i.e., line profile 2) facets. The line profiles confirm the absence of segregation at the surface.

**Figure 12:**
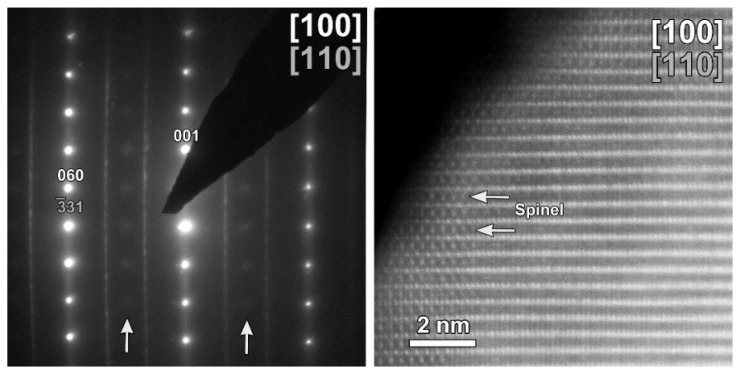
HAADF-STEM images of the cycled CP-NMC sample along the [100]/[110] orientation, showing a significant structure degradation.

The same TEM study was performed on the cycled CP-NMC sample (Figure 11). Similar to the SG-NMC sample, diffuse intensities are present in the SAED pattern, indicating the formation of a spinel-type phase. The corresponding HAADF-STEM image confirms that the surface of the particle has partially degraded to a spinel-type phase upon cycling. Furthermore, the EDX map and the line profiles taken from the (200) and (002) facets show the absence of segregation at the surface. We can conclude that structure degradation is present in both samples, but it is more significant in the CP-NMC sample. This is clearly visible in the HAADF-STEM image taken along the [100]/[110] orientation of the CP-NMC sample, shown in Figure 12, which shows a significant formation towards a spinel-type phase at the surface. Such a high degree of structure degradation was only observed in the CP-NMC sample, not in any particle of the SG-NMC sample.

## 4. Discussion

The TEM study showed that the particle size distribution for the SG-NMC sample is more uniform than for the CP-NMC sample and that the particles prepared by the solution-gel method are much smaller than the particles obtained by the co-precipitation method. In addition, we found that the SG-NMC sample displays a significant capacity decay compared to CP-NMC, but that both samples are subjected to voltage decay. The occurrence of voltage fade is delayed in the case of the SG-NMC sample, resulting in an overall lower voltage fade compared to CP-NMC. Our results for the particle size and electrochemical properties are consistent with the study of Santhanam et al. [22] where the morphology of the Li_1.05_Ni_1/3_Mn_1/3_Co_1/3_O_2_ sample prepared by an SG and CP route is compared. Santhanam et al. suggest that for nanoparticles, the rate capability is expected to improve due to the higher surface area for charge transfer and the shorter diffusion length. On the other hand, the high surface area implies a high surface reactivity, which can lead to an increase in detrimental surface side-reactions and poor inter-particle electrical contact deteriorating the capacity retention at the high C-rates (1C and 8C) reported by Santhanam et al. More specifically, at the particle’s surface, electrolyte decomposition originated from the reaction of the solvent (EC:DMC in our case) and oxygen originated from the active material results in the formation of an insulating surface film (CEI; cathode electrolyte interphase), as based on Santhanam et al. Downsizing particles results in a higher overall surface area, implying a larger content of insulating surface film formation. As these insulating surface films hamper the contact between the active material and electrolyte increasing the charge transfer resistance, the capacity is penalized. In addition, a larger overall surface area of the NMC active material could result in a larger extent of transition metal dissolution by HF etching. HF is formed by the reaction of the LiPF_6_ electrolyte salt with trace impurities of H_2_O present in the electrolyte. Our SG-NMC sample delivers a slightly higher capacity in the first cycle, which can be ascribed to the higher surface area; however, the capacity drastically decreases during extended cycling, which is not the case in our CP-NMC sample. The good capacity retention in the latter sample can be partially attributed to the decreased side-reactions with the electrolyte, which are related to the lower surface area.

The TEM study also revealed structural modifications at selective surface planes in the case of the SG-NMC sample, where a Ni-rich surface layer is present at the {200} facets that adopts a spinel-type structure, while a Co-rich surface layer is observed at the {002} and {202} facets, but not at the {$20\bar{2}$} facets. Both the {202} and {$20\bar{2}$} facets tend to form a rock-salt-type structure, showing that the Co-segregation is independent of the formation of the rock-salt-type structure. The TEM study on the cycled SG-NMC sample showed a reduced degradation on the {200} facets. As the spinel-type structure is highly stable, the Ni-rich spinel-type layer can also be considered as a protective surface layer that slows down surface corrosion and structure degradation. However, the SAED pattern of the cycled SG-NMC sample clearly shows diffuse reflections that indicate the formation of a significant amount of spinel-type phase. Therefore, we can conclude that the spinel-type surface layer might act as a protecting layer to inhibit structural degradation at that specific facet, but that it is unable to significantly reduce the structural degradation of the entire particle, as supported by the diffuse intensities in the SAED pattern of SG-NMC. This might explain why the SG-NMC sample is less degraded after 50 cycles than the CP-NMC sample, resulting in a reduced voltage fade as observed for the SG-NMC sample. During the formation of the spinel, oxygen loss will occur, as the ratio between the transition metals and oxygen is 2:4 (TM:O) instead of 2:5 in Li_1.2_(TM)_0.8_O_2_, which is partially responsible for the voltage plateau. The voltage plateau of the CP-NMC sample is slightly larger than that of the SG-NMC sample, confirming our conclusion.

In addition to what was found by Santhanam et al. [22], it can be concluded that not only particle size but also surface modifications contribute to electrochemical performance. The segregation of Ni and Co at selective surface facets of Li_1.2_Ni_0.13_Mn_0.54_Co_0.13_O_2_ agrees with that reported by Yan et al. [23]. Similar to our results, Yan et al. found that Ni is exclusively segregated at the {200} surfaces and adopts a spinel-type structure, while Co predominately enriches the {002} facets with a rock-salt-type structure. However, in their case, they also observed a Co-enriched surface layer at the {$20\bar{2}$} facets, whereas in our case, the rock-salt-type surface layer was not pronouncedly Co-rich. In the literature, the appearance of Ni- and Co-segregation was also observed for samples prepared by the CP method [21,23], while our results show that it only occurs for the particles synthesized by our SG synthesis route. In our study, carbonate co-precipitation was used, while the syntheses in references [21,23] are based on hydroxide co-precipitation, which might be responsible for the differences in the obtained results. In the case of hydroxide co-precipitation synthesis, Zheng et al. [21] and Yan et al. [23] state that Ni and Co surface segregation can be inhibited by increasing the elemental Ni:Mn ratio. When the elemental ratio of both transition metals is equal to 1, exclusively Ni surface segregation is obtained. No surface segregation is observed for Ni-rich NMC. When performing hydroxide co-precipitation synthesis at pHs between 9 and 11, using ammonia as base, nickel and cobalt hydroxides will preferentially redissolve under the form of ammine complexes and subsequently recrystallize, as reported by van Bommel et al. [24] and Shen et al. [25] The stability of manganese ammine complexes at these pHs is too low to facilitate noteworthy redissolution from precipitated manganese hydroxides. As such, the surfaces of the intermediates after hydroxide co-precipitation will be surface enriched with Ni and Co, facilitating the formation of Ni and Co surface segregation at facets after the subsequent thermal treatment. At pHs above 11 redissolution and recrystallization of nickel species will be dominant, resulting in Ni-surface enrichment. The low stability of manganese ammine complexes at basic pHs puts serious constraints on the hydroxide co-precipitation synthesis of Mn-rich NMCs having a uniform transition metal stoichiometry without surface enrichment. Hou et al. [26] showed that a way to compensate for the lower complexing ability of Mn^2+^ with ammonia at basic pHs is to use dual ammonia-oxalate chelating agents, as oxalate shows a higher chelating ability with Mn^2+^ cations. The proposed synthesis route initially comprises the formation of dual ammine-oxalate transition metal complexes, subsequently precipitated as transition metal hydroxides via an anion-exchange mechanism. In the frame of the dissolution-recrystallization mechanism, the higher ability of oxalate to form Co(II) and Mn(II) oxalate complexes, most probably compensates for the higher stability of nickel ammine complexes at pHs above 9 compared to their cobalt and manganese ammine counterparts. No experimental evidence in the literature is available that demonstrates that the absence of sufficient dissolution-recrystallization above certain pHs in the presence of aqueous NH_3_ is directly correlated with Ni and Co surface segregation in the final oxide end product obtained after thermal treatment. The absence of Ni and/or Co surface segregation for carbonate co-precipitation synthesis as shown in this work and the presence of hydroxide co-precipitation synthesis as reported in the references discussed above can be potentially, at least partially, correlated with different tendencies of transition metal species to dissolve and recrystallize and/or with the given that (Ni, Mn, Co) carbonates precipitate simultaneously. For our carbonate co-precipitation route, performed at a controlled pH of 7.5, the transition metals will most probably redissolve as ammine complexes and potentially recrystallize. At higher pHs between about 8.5 and 9.8, the nickel ammine complexes will be very stable, resulting in a transition metal non-stoichiometry similar to the hydroxide co-precipitation case discussed above [27]. Xiang et al. [28] showed that at pH = 7.5, nickel and cobalt also tend to precipitate as basic carbonates as Ni_2_(OH)_2_CO_3_ and Co_2_(OH)_2_CO_3_, respectively. Those basic carbonates are thermodynamically more stable than their carbonate counterparts and by consequence the equilibrium for the reaction (1) below will be more shifted to the M_2_(OH)_2_CO_3_ (M=Ni, Co) side.

(1) $$M_{2}{\left(\mathrm{OH}\right)}_{2}{\;\mathrm{CO}}_{3}+2{\mathrm{nNH}}_{3}⇌2{\left[M{\left({\mathrm{NH}}_{3}\right)}_{n}\right]}^{2+}+2{\mathrm{OH}}^{-}+{\mathrm{CO}}_{3}^{2-}$$

As such, at least a part of the lower stability of ammine complexes for manganese at pH = 7.5 will be compensated by the higher stability of nickel and cobalt basic carbonates compared to Mn carbonates. In general, it can be concluded that because of the cobalt and nickel basic carbonate formation at a pH close to neutral, hydroxide co-precipitation synthesis is preferred over carbonate co-precipitation synthesis for Ni-rich NMCs while the opposite is valid for Mn-rich NMCs.

Besides the segregation of the TM cations of Ni and Co at selective crystal surfaces [23,29,30] different studies have also reported Ni-segregation at the domain boundaries between orientation variants in pristine materials [31,32] with the width of the segregation domains increasing upon cycling. Jarvis et al. [32] propose that the Ni-rich domain boundaries reduce the rate of the Li diffusion and could contribute to the overall capacity fade. In our study, the severe capacity decay observed for SG-NMC has to have a different origin, as we have not observed any Ni-rich region.

## 5. Conclusions

This study showed that the method used to synthesize Li_1.2_Ni_0.13_Mn_0.54_Co_0.13_O_2_ particles strongly affects the electrochemical performance as, for example, the sample prepared by the solution-gel method exhibits a significant capacity decay compared to the sample prepared by the co-precipitation method. On the other hand, the voltage of the SG-NMC sample is slightly more stable upon cycling than that of the CP-NMC sample. Based on the TEM study, we could conclude that the higher capacity fade observed in the SG-NMC sample is most likely attributed to the smaller particle size, but that the Ni-enriched spinel-type surface layer present on the {200} facets of the SG-NMC sample slows down the structural degradation at these specific facets, resulting in a lower voltage fade compared to the CP-NMC sample.

## Data Availability

Not applicable.

## Supplementary Materials

The following supporting information can be downloaded at: https://www.mdpi.com/article/10.3390/nano12132269/s1, Figure S1: SEM images of the SG-NMC (left) and CP-NMC (right) samples; Figure S2: XRPD patterns of the CP-NMC (top) and SG-NMC (bottom) samples; Figure S3: SAED and HAADF-STEM images of the SG-NMC sample; Figure S4: SAED and HAADF-STEM images of the CP-NMC sample; Figure S5: HAADF-STEM image of the (200) facet taken from the particle shown in Figure 2 before and after the EDX acquisition; Figure S6: HAADF-STEM image with the mixed (Co, Ni, Mn) element map taken from the particle shown in Figure 2 in the main text from the first batch.
