# Supplementary material for: The Influence of Synthesis Method on the Local Structure and Electrochemical Properties of Li-Rich/Mn-Rich NMC Cathode Materials for Li-Ion Batteries
Source: Nanomaterials (Basel). 2022 Jun 30;12(13):2269. doi: 10.3390/nano12132269 (PMC9268383; doi:10.3390/nano12132269)
Supplement: Supplementary file 1 [file nanomaterials-12-02269-s001.zip › nanomaterials-1777496-SI.pdf]

The Influence of Synthesis Method on the Local Structure and Electrochemical Properties of Li-Rich/Mn-Rich NMC Cathode Materials for Li-Ion Batteries

Mylène Hendrickx [1], Andreas Paulus [2], Maria A. Kirsanova [3], Marlies K. Van Bael [2], Artem M. Abakumov [3], An Hardy [2] and Joke Hadermann [1,*]

[1] EMAT, Department of Physics, University of Antwerp, Groenenborgerlaan 171, 2020 Antwerp, Belgium; mylene.hendrickx93@gmail.com

[2] DESINe Group, Materials Chemistry, Institute for Materials Research (IMO-Imomec), Hasselt University, Agoralaan Building D and Imec, Division Imomec, and Energyville, 3590 Diepenbeek, Belgium; andreas.paulus@uhasselt.be (A.P.); marlies.vanbael@uhasselt.be (M.K.V.B.); an.hardy@uhasselt.be (A.H.)

[3] Skolkovo Institute of Science and Technology, Center for Energy Science and Technology, Nobel Str 3, Moscow 121205, Russia; maria.kirsanova@skoltech.ru (M.A.K.); a.abakumov@skoltech.ru (A.M.A.)

* Correspondence: joke.hadermann@uantwerpen.be

Supporting information

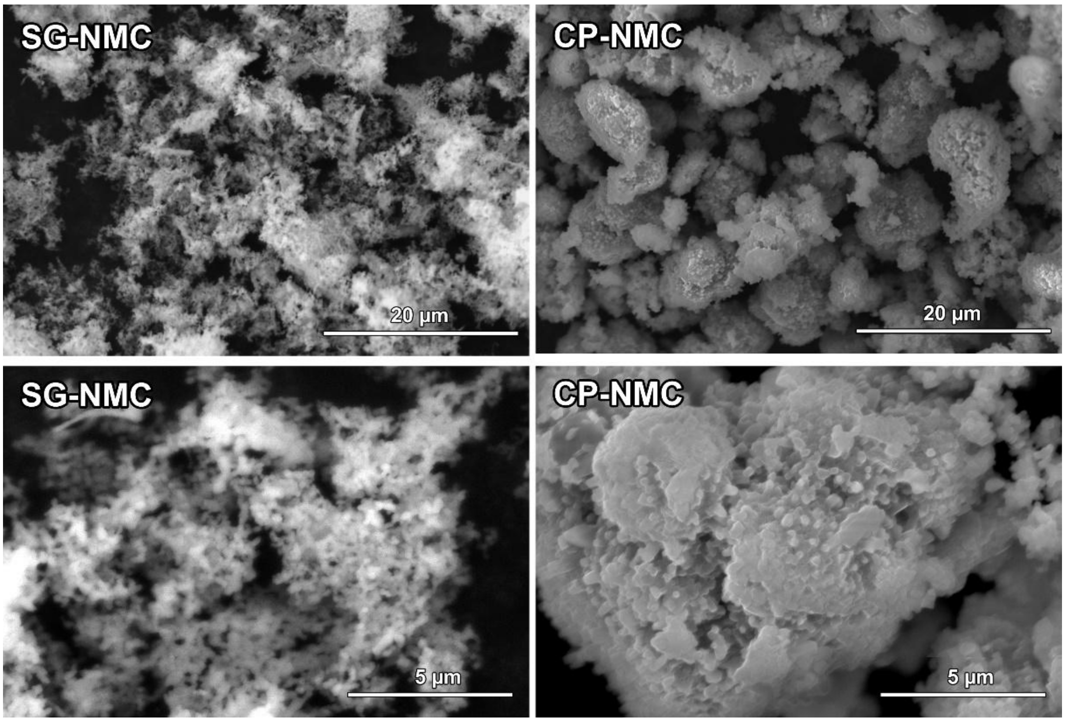

Figure S1. SEM images of the SG-NMC (**left**) and CP-NMC (**right**) samples.

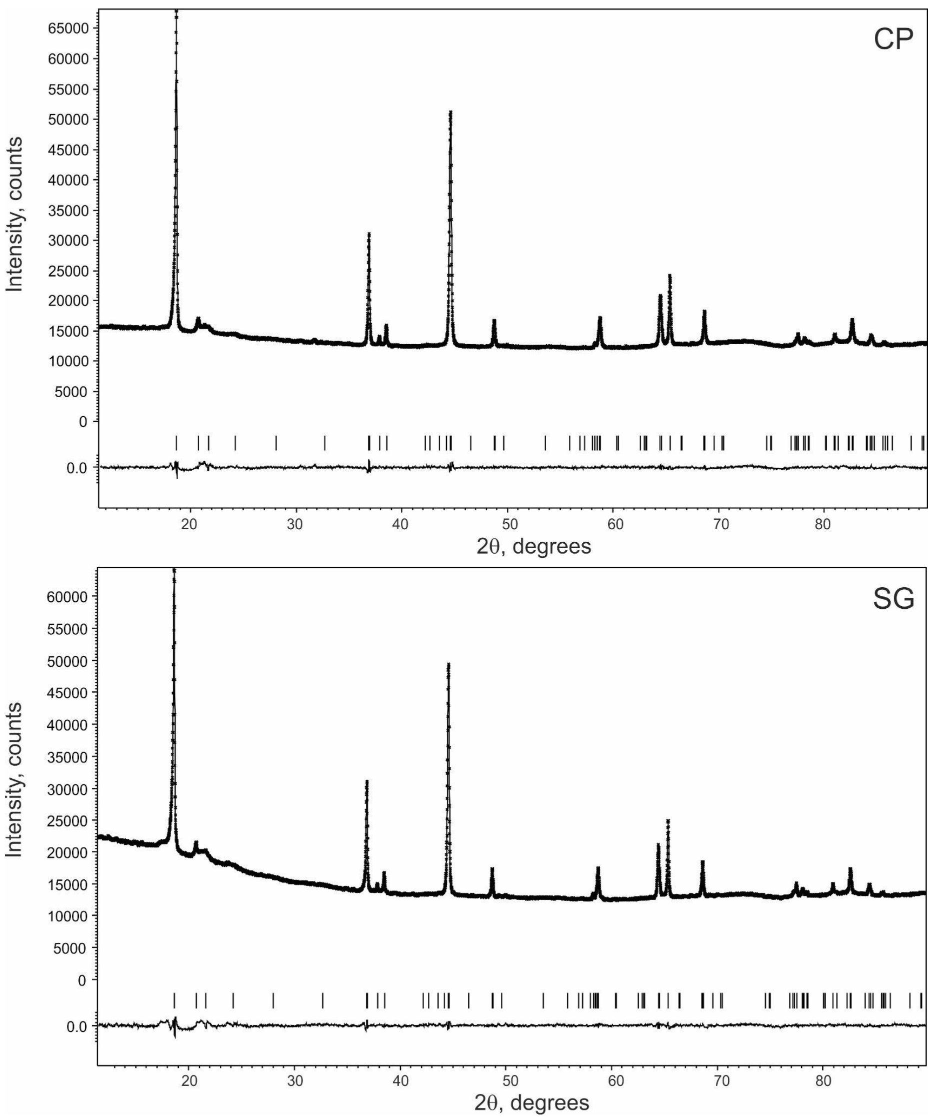

Figure S2. XRPD patterns of the CP-NMC (**top**) and SG-NMC (**bottom**) samples after fitting with the LeBail method. For both samples, no additional phases are observed in the XRPD patterns, which means that the Co-rich additional phase present in the SG-NMC sample has a concentration of less than 5%. The vertical bars mark the reflection positions corresponding to the monoclinic *C2/m* unit cell with the parameters a=4.93982(7)Å, b=8.5511(1)Å, c=5.02586(8)Å, β=109.256(1)°, V=200.421(5)Å^3 for the CP-NMC sample and a=4.94145(8)Å, b=8.5554(1)Å, c=5.02675(7)Å, β=109.243(1)°, V=200.639(5)Å^3 for the SG-NMC sample.

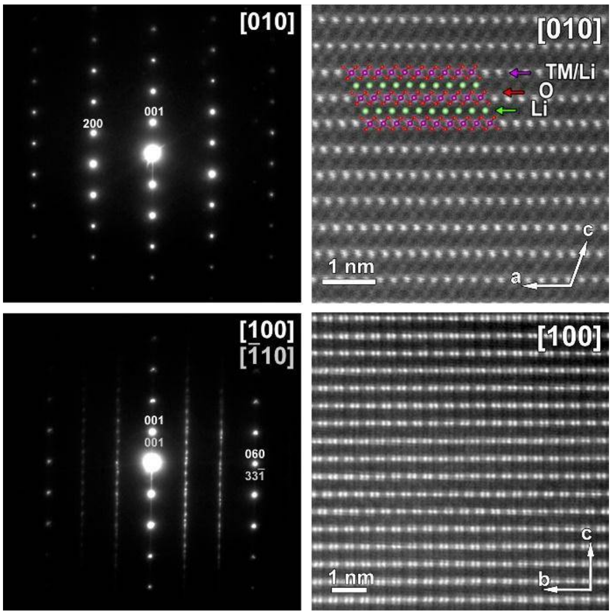

Figure S3. SAED and HAADF-STEM images of the SG-NMC sample. Top: the SAED pattern (**left**) and HAADF-STEM image (**right**) along the [010] orientation showing the layered structure with O3-stacking. Bottom: the SAED pattern (**left**) and HAADF-STEM image (**right**) along the [100] orientation showing the honeycomb ordering within the mixed Li-TM layers which is demonstrated by a prominent pattern of pairs of bright dots (i.e. atom columns of transition metals) and less bright dots (i.e. atom column containing both Li and transition metals) in between.

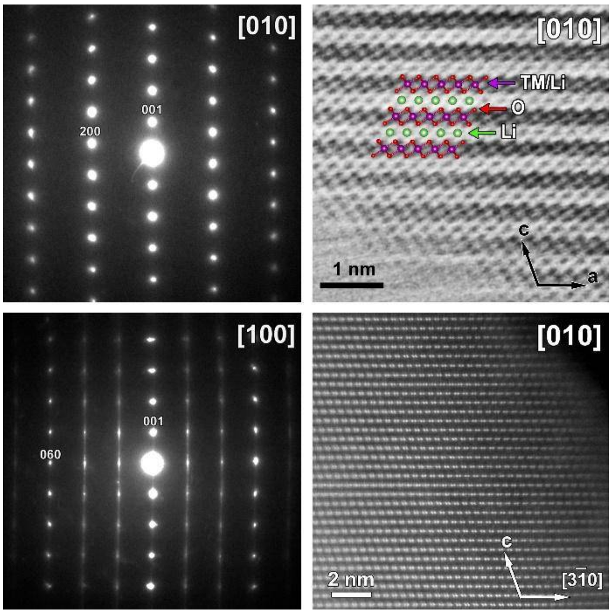

Figure S4. SAED and HAADF-STEM images of the CP-NMC sample. Top: the SAED pattern (**left**) and HAADF-STEM image (**right**) along the [010] orientation showing the layered structure with O3-stacking. Bottom: the SAED pattern (**left**) and HAADF-STEM image (**right**) along the [100] and [110] orientation, respectively, showing the honeycomb ordering within the mixed Li-TM layers which is demonstrated by a prominent pattern of pairs of bright dots (i.e. atom columns of transition metals) and less bright dots (i.e. atom column containing both Li and transition metals) in between.

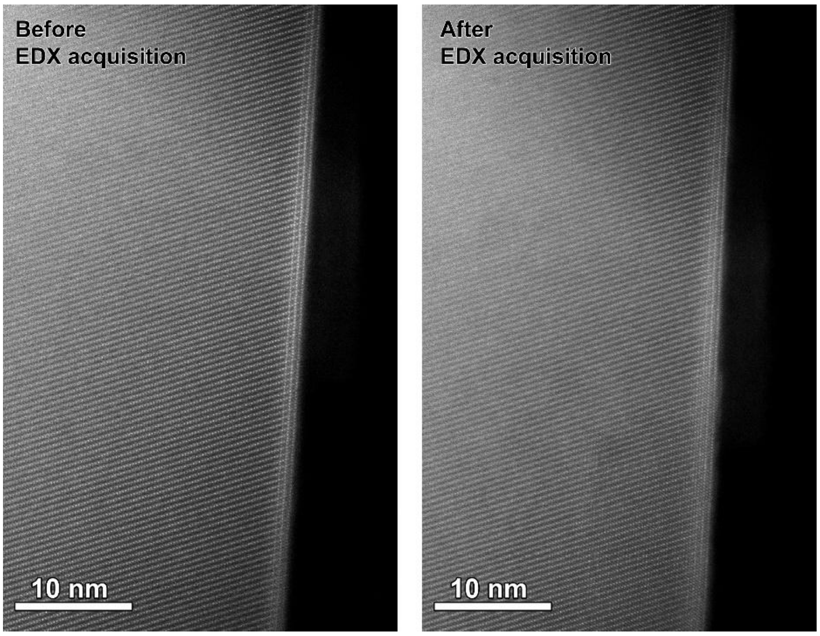

Figure S5. HAADF-STEM image of the (200) facet taken from the particle shown in Figure 2 before and after the EDX acquisition. The increased intensity at the (200) facet, which corresponds to the Ni enrichment at the surface, is clearly visible before the EDX acquisition, showing that the Ni-segregation is not induced by the long exposure of the electron beam during the EDX acquisition.

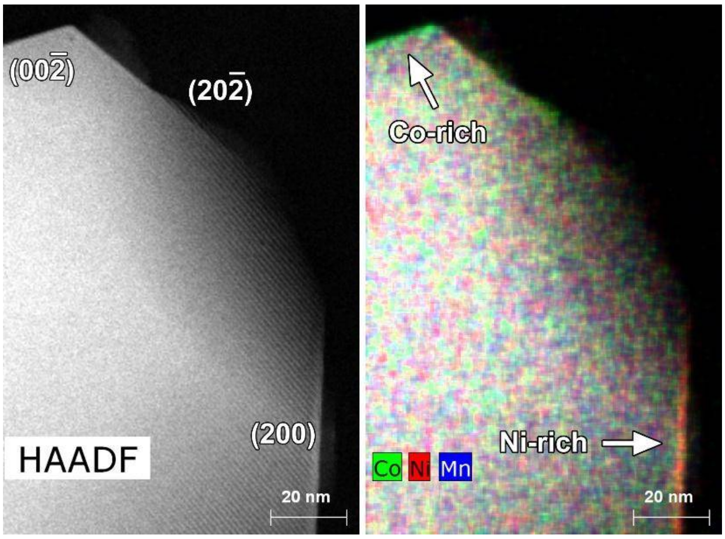

Figure S6. HAADF-STEM image with the mixed (Co, Ni, Mn) element map taken from the particle shown in Figure 2 in the main text from the first batch, showing that the $(20\underline{2})$ facet has almost no Co-segregation.
